# Troponin and Anti-Troponin Autoantibody Levels in Patients with Ventricular Noncompaction

**DOI:** 10.1371/journal.pone.0057648

**Published:** 2013-02-28

**Authors:** Hatice Betül Erer, Tolga Sinan Güvenç, Ahu Sarbay Kemik, Hale Yaka Yılmaz, Şeref Kul, Servet Altay, Nurten Sayar, Yüksel Kaya, Mehmet Eren

**Affiliations:** 1 Dr. Siyami Ersek Cardiovascular and Thoracic Surgery Research and Training Hospital, Department of Cardiology, İstanbul, Turkey; 2 Kafkas University School of Medicine, Department of Cardiology, Kars, Turkey; 3 Istanbul University Cerrahpaşa Faculty of Medicine, Department of Biochemistry, İstanbul, Turkey; 4 Bezmialem Vakıf University, Faculty of Medicine, İstanbul, Turkey; Tokai University, Japan

## Abstract

Ventricular hypertrabeculation/noncompaction is a morphologic and functional anomaly of myocardium characterized by prominent trabeculae accompanied by deep recessus. Dilated cardiomyopathy with left ventricular failure is observed in these patients, while the cause or pathophysiologic nature of this complication is not known. Anti-troponin antibodies are formed against circulating cardiac troponins after an acute coronary event or conditions associated with chronic myocyte necrosis, such as dilated cardiomyopathy. In present study, we aimed to investigate cardiac troponins and anti troponin autoantibodies in ventricular noncompaction/hypertrabeculation patients with/without reduced ejection fraction. A total of 50 patients with ventricular noncompaction and 23 healthy volunteers were included in this study. Noncompaction/hypertrabeculation was diagnosed with two-dimensional echocardiography using appropriate criteria. Depending on ejection fraction, patients were grouped into noncompaction with preserved EF (LVEF >50%, n = 24) and noncompaction with reduced EF (LVEF <35%, n = 26) groups. Troponin I, troponin T, anti-troponin I IgM and anti-troponin T IgM were measured with sandwich immunoassay method using a commercially available kit. Patients with noncompaction had significantly higher troponin I (28.98±9.21 ng/ml in NCNE group and 28.11±10.42 ng/ml in NCLE group), troponin T (22.17±6.97 pg/ml in NCNE group and 22.78±7.76 pg/ml in NCLE group) and antitroponin I IgM (1.92±0.43 µg/ml in NCNE group and 1.79±0.36 µg/ml in NCLE group) levels compared to control group, while antitroponin T IgM and IgG were only elevated in patients with noncompaction and reduced EF (15.81±6.52 µg/ml for IgM and 16.46±6.25 µg/ml for IgG). Elevated cardiac troponins and anti-troponin I autoantibodies were observed in patients with noncompaction preceding the decline in systolic function and could indicate ongoing myocardial damage in these patients.

## Introduction

Venricular hypertrabeculation/noncompaction (NC/HT) is a primarily genetic cardiomyopathy characterized by prominent trabeculae with deep recessus separating trabeculations [1]. Left ventricular systolic dysfunction and development of idiopathic cardiomyopathy are the most important consequences of NC/HT [1], [2]. However, not all patients with NC/HT demonstrate left ventricular failure, and some patients may remain asymptomatic for long periods [3]. The cause for transformation to a dilated cardiomyopathy (DCM) phenotype remains unknown despite being a topic of active research.

Both cardiac troponin I (cTnI) and cardiac troponin T (cTnT) are components of myocardial troponin-tropomyosin complex and elevated serum levels are observed in dilated cardiomyopathy (DCM) patients due to myocardial necrosis, apoptosis or myocardial leakage. Elevated troponin levels are almost invariably related with poor prognosis in DCM patients [4], [5]. Antibodies against various myocardial components, including cardiac troponins, were observed in patients with left ventricular systolic dysfunction and in normal individuals [6], [7]. Of those, anti-cTnI antibodies were shown to be elevated in patients with idiopathic dilated cardiomyopathy (iDCM) and ischemic cardiomyopathy compared to health controls [8]. Animal studies had demonstrated that autoantibodies against cTnI could alter calcium currents in mice and produce cardiac lesions similar to the ones observed in iDCM [9]. While the evidence does not definitely demonstrate a role for autoimmunity in the development of DCM, it is hypothesized that immunization against myocardial compartments could accentuate cardiac dysfunction.

While a few studies and report had shown elevated cTnT levels in NC/HT patients with accompanying neuromuscular disorders [10], a detailed investigation regarding to troponin and antitroponin values in NC/HT patients is missing. In this study, we aimed to measure serum troponin I and T, as well as antitroponin I and T levels in a cohort of NC/HT patients with and without left ventricular systolic dysfunction.

## Materials and Methods

### Patient Selection

A total of 50 patients with a previous diagnosis of NC/HT and followed up by institutional heart failure and cardiac transplantation clinic between years 2006 and 2012 were included in this study. All enrolled patients were re-evaluated to confirm previous diagnosis of NC/HT as detailed below. Patients who had severe aortic stenosis or obstructive lesions at left ventricular outflow tract, complex congenital heart lesions, had existing diseases that might cause left ventricular dysfunction or other forms of cardiomyopathy, and patients with previous myocardial infarction were excluded from study. Patients with left ventricular noncompaction were separated in two groups based on their ejection fraction: those with a left ventricular EF of less than 35% (NCLE group) and those with a left ventricular EF of equal to or more than 50% (NCNE group). Additionally, twenty-three volunteers without known coronary artery disease or previous myocardial infarction and had an ejection fraction equal to or more than 50% served as healthy controls.

For all subjects, demographic data were collected after inclusion to study. This study was approved by Dr. Siyami Ersek Hospital ethics committee and all patients gave their written informed consent. For patients below 18 years, a written consent was obtained from legal guardians.

### Echocardiographic Examination

**Figure 1:**
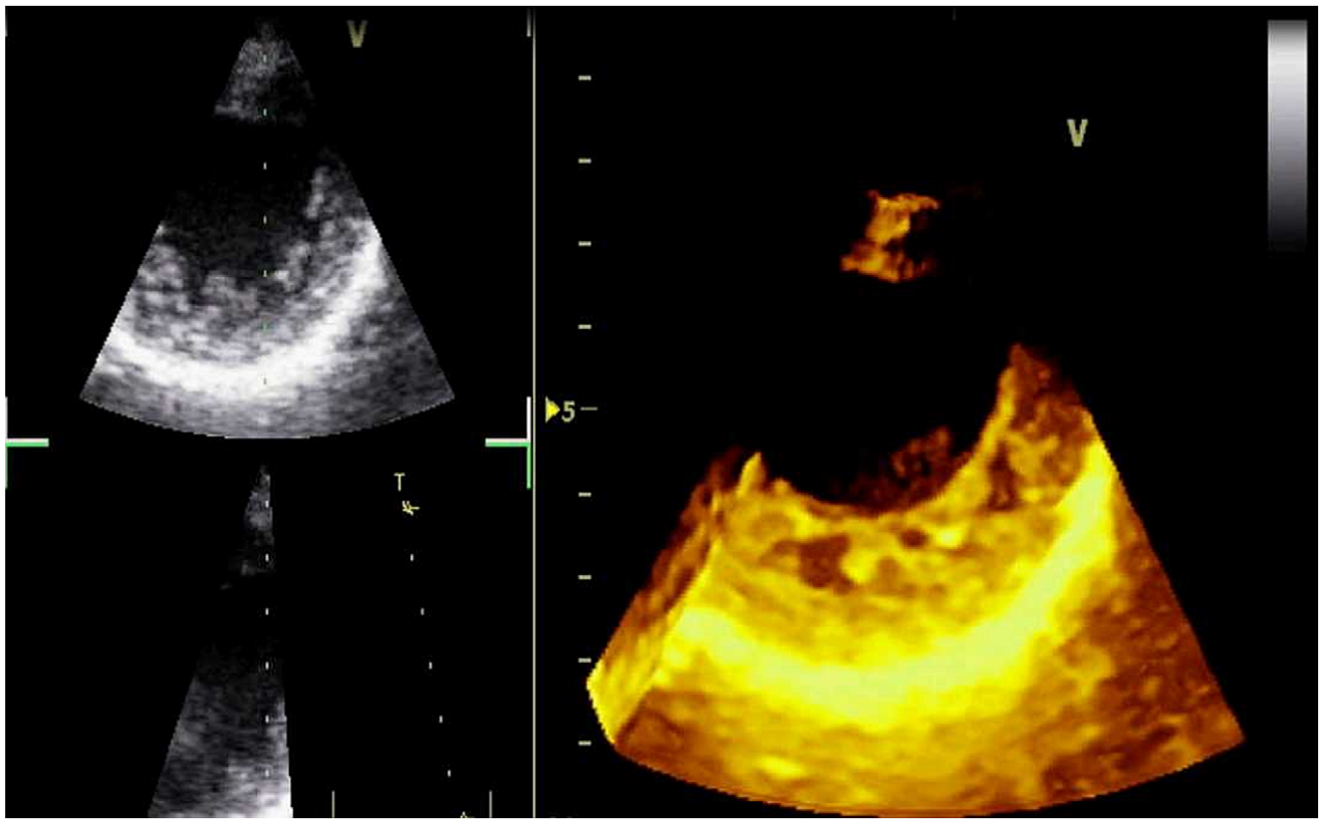
Real-time three-dimensional echocardiographic appearance from parasternal short-axis view of a patient with isolated left ventricular noncompaction.

Echocardiographic examination was performed with an echocardiography platform (GE Vivid 7, GE Healthcare, Piscataway, New Jersey, USA) equipped with a 1.5–3.6 MHz phased-array transducer. Diagnosis of noncompaction was established when all of these conditions were satisfied from parasternal short-axis view: observation of three or more prominent trabeculations, deep recessus in contact with blood, and a noncompacted-to-compacted myocardium of more than 2 during systole [10], [11] (Figure 1). For all patients, the diagnosis was confirmed by two cardiologists with experience in transthoracic echocardiography (HBE and NS). End-diastolic and end systolic volume of left ventricle, as well as left ventricular ejection fraction were calculated using biplane Simpson method from apical four-chamber and two chamber views. Number of segments with hypertrabeculation/noncompaction was calculated from parasternal short axis views at basal, mid and apical sections of left ventricle using 16-segment model. Mitral E and A values were measured from the tips of mitral leaflets with pulsed-wave Doppler in apical 4-chamber view. Isovolumic contraction time (IVCT) and isovolumic relaxation time (IVRT) were measured with continuous-wave (CW) Doppler with cursor aligned to record both mitral inflow and aortic outflow velocities from apical long axis view. Aortic ejection time (AET) was measured from aortic outflow recordings taken with CW Doppler from apical long axis view. Early diastolic velocity of mitral annulus (Em) was measured from the lateral aspect of mitral valve with TDI from apical 4-chamber view. Left ventricular myocardial performance index was calculated as (IVCT+IVRT)/AET. For all enrolled subjects, a detailed echocardiogram was carried out to rule out other types of cardiomyopathy, more than mild valvular diseases or congenital defects.

### Blood Withdrawal and Analysis

Patients and healthy controls were recalled one day after echocardiographic examination with overnight fasting before collection of blood samples. Whole blood samples were obtained from subjects in the supine position from the antecubital vein with a 20-gauge needle by applying minimal tourniquet force. For all subjects, 8 cc blood was collected in a dry tube and measurements for cTnI, cTnT, antitroponin I IgM, antitroponin T IgM and antitroponin T IgG were made as detailed below. The laboratory was blinded for samples.

### Measurement for Cardiac Troponins

A serum separator tube was used and samples were allowed to clot for 30 minutes before centrifugation for 20 minutes at approximately 1000×g. Samples were then stored at a temperature between −20° and −80°C. Design of the assay was based on a sandwich enzyme linked immunosorbent assay (ELISA) using Dimension analyzer (Siemens Healthcare, Erlangen, Germany). The microtiter plate provided in the kit had been precoated with a monoclonal antibody specific to human troponin I or troponin T. Samples were pippetted into these wells. Unbound troponin and other components of the sample were removed by washing and biotin conjugated monoclonal antibody (Biovendor, Heidelberg, Germany) specific to troponin I or troponin T was added. To quantitatively determine the amount of troponin I or troponin T present in the sample, avidin conjugated to horseradish peroxidase was then added to each microplate well. Next, a TMB-substrate solution was added to each well. Finally, a sulfiric acid solution was added and a resulting yellow colored product was formed. The optical density of wells was determined using a microplate reader set to 450 nm. The absorbance is directly proportional to the amount of captured troponin I or troponin T.

### Measurement for Antitroponin I or Antitroponin T

The samples were kept at room temperature for 20 minutes to allow clotting, were centrifuged, and were then stored at −20 degrees. The values listed of marker represent the peak concentrations during each collection period. A sandwich enzyme immunologic assay (ELISA) method was used with a Dimension analyzer (Siemens Healthcare, Erlangen, Germany). Based on a technique using the streptavidin, the single step sandwich assay allowed serial determination of blood samples to be made within 2 hours. The test was carried out in microprocessor-controlled photometers and required streptavidin-coated tubes and two monoclonal antihuman cardiac anti troponin T and anti troponin I antibodies (Biovendor, Heidelberg, Germany). During a 60 minute incubation period, the antigen was bound by one biotinylated and one peroxidase-labeled antibody. This complex adhered to the test tube wall because of the high affinity streptavidin-biotin interaction. After two washing steps, the substrate chromogen was added.

### Statistical Analysis

Statistical analysis was performed using SPSS 17.0 (IBM, Armonk, NY). Quantitative data was given as mean ± SD and categorical data was given as percentages. Distribution of scalar data was determined with Kolmogorov-Smirnov test. For scalar variables, comparisons between groups were performed with one-way ANOVA and post-hoc Scheffe test or Kruskal-Wallis and Mann-Whitney U tests, as appropriate. For categorical variables, χ^2^ and Fisher’s exact tests were used. Correlation analyses were performed using Spearman’s rho. A p value of <0.05 was accepted as significant.

## Results

Demographic and clinical data, including previous medications and interventions regarding to study groups are given in Table 1. There were no significant difference between patients regarding to age, gender, history of hypertension, history of diabetes, smoking habits and family history of cardiomyopathy. Arrhythmic events were observed significantly higher in patients with noncompaction compared to controls. Left ventricular end diastolic and end systolic volumes were higher in NCLE group compared to other groups (170.25±40.86 ml vs. 140.99±53.05 ml and 97.35±26.75 ml; p<0.05 for LVEDv and 121.45±32.01 ml vs. 71.58±46.49 ml and 27.45±7.46 ml; p<0.001 for LVESv), and in NCNE group compared to healthy controls (p<0.01 for LVEDv and p<0.001 for LVESv). Left ventricular ejection fraction was significantly lesser in NCLE group (28.08% ±7.08%) compared to NCNE (56.87% ±6.05%) group and healthy volunteers (61.82% ±3.28%, p<0.001) and also significantly lower in NCNE group compared to controls (p<0.01). Ratio of mitral E wave to A wave was similar between groups, while ratio of mitral E wave to Em was significantly higher in NCLE group (p<0.05). Left ventricular myocardial performance index was significantly higher in NCLE group compared to both groups (p<0.001). Number of segments with hypertrabeculation/noncompaction was significantly higher in NCLE group (4.05±1.13) compared to NCNE group (2.44±0.62; p<0.001) (Table 2).

**Table 1:** Demographic and clinical variables regarding to study groups.

Parameter	Normal Controls (n = 23)	Noncompaction with Normal EF (n = 24)	Noncompaction with Reduced EF (n = 26)	P Value
**Demographic and Clinical Variables**				
**Age (yr)**	34±10.0	36±16.8	37±16.2	NS
**Gender (%Male)**	44%	42%	80%	NS
**Hypertension (%)**	17%	37%	19%	NS
**Diabetes (%)**	4%	4%	15%	NS
**Smoking (%)**	22%	13%	19%	NS
**Family History for Cardiomyopathy (%)**	0%	17%	12%	NS
**NYHA Class**				
**Class I**			3.8%	
**Class II**	(−)	(−)	19.2%	(−)
**Class III**			53.8%	
**Class IV**			23.1%	
**Supraventricular or Ventricular Arrhythmias**	0%	21%*	23%*	**<0.05**
**Heart Rate (bpm)**	76.61±6.81	72.29±14.44	81.42±15.21*	**<0.05**
**Systolic Blood Pressure (mmHg)**	121.83±11.86	126.88±11.33*	115.46±12.76	**<0.01**
**Diastolic Blood Pressure (mmHg)**	73.52±8.13	76.33±8.24*	68.96±8.78	**0.01**
**Previous Drug Usage and Interventions**				
**ACE Inhibitor/ARB (%)**	13%	42%*	92%**	**<0.001**
**Calcium Channel Blocker (%)**	10%	17%	8%	NS
**Beta Blocker (%)**	4%	54%*	92%**	**<0.001**
**Amiodarone (%)**	0%	8%	27%*	**<0.05**
**Digoxin (%)**	0%	0%	46%**	**<0.001**
**Diuretics (%)**	0%	4%	88%**	**<0.001**
**Antiplatelet or Anticoagulant Therapy (%)**	9%	25%	88%**	**<0.001**
**Implantable Cardioverter Defibrillator (%)**	0%	8%	12%	NS
**Cardiac Resynchronization Therapy (%)**	0%	0%	4%	NS

Data is given ± SD for scalar variables. ACE, angiotensin converting enzyme; ARB, angiotensin receptor blocker. Provided p value refers to significance level for comparisons between all groups.

* Significantly higher compared to lowest values.

** Significantly higher compared to all groups.

**Table 2:** Echocardiographic and laboratory variables regarding to study groups.

Parameter	Normal Controls(n = 23)	Noncompaction withNormal EF (n = 24)	Noncompaction withReduced EF (n = 26)	P value
**Echocardiographic Variables**				
**LVEDD (mL)**	97.35±26.75	140.99±53.05**	170.25±40.86**	**<0.001**
**LVESD (mL)**	27.45±7.46	71.58±46.49*	121.45±32.01**	**<0.001**
**LVEF (%)**	61.83±3.28**	56.88±6.05*	28.08±7.08	**<0.001**
**Mitral E/A**	1.37±0.20	1.36±0.39	1.37±0.91	NS
**Mitral E/Em**	5.78±1.49	6.43±1.28	10.8±6.35	**<0.05**
**LV MPI**	0.45±0.06*	0.39±0.21	0.73±0.14**	**<0.001**
**Number of Segments with NC/HT**	(−)	2.44±0.62	4.05±1.13**	**<0.001**
**Laboratory Analysis**				
**Troponin I (ng/ml)**	9.74±1.97	28.98±9.21*	28.11±10.42*	**<0.001**
**Troponin T (pg/ml)**	13.42±4.19	22.17±6.97*	22.78±7.76*	**<0.01**
**Troponin I IgM Antibodies (µg/ml)**	0.80±0.23	1.92±0.43*	1.79±0.36*	**<0.001**
**Troponin T IgM Antibodies (µg/ml)**	12.00±2.32	12.33±5.68	15.81±6.52**	**<0.05**
**Troponin T IgG Antibodies (µg/ml)**	11.91±2.33	12.34±5.80	16.46±6.25**	**0.01**

Data is given ± SD for scalar variables. LVEDD, Left ventricular end diastolic volume; LVESD, Left ventricular end systolic volume; LVEF, Left ventricular ejection fraction; E/A, ratio of mitral early flow to atrial flow; E/Em, ratio of mitral early flow to early diastolic movement of mitral annulus; NC/HT, hypertrabeculation/noncompaction. Provided p value refers to significance level for comparisons between all groups.

* Significantly higher compared to lowest values.

** Significantly higher compared to all groups.

**Figure 2:**
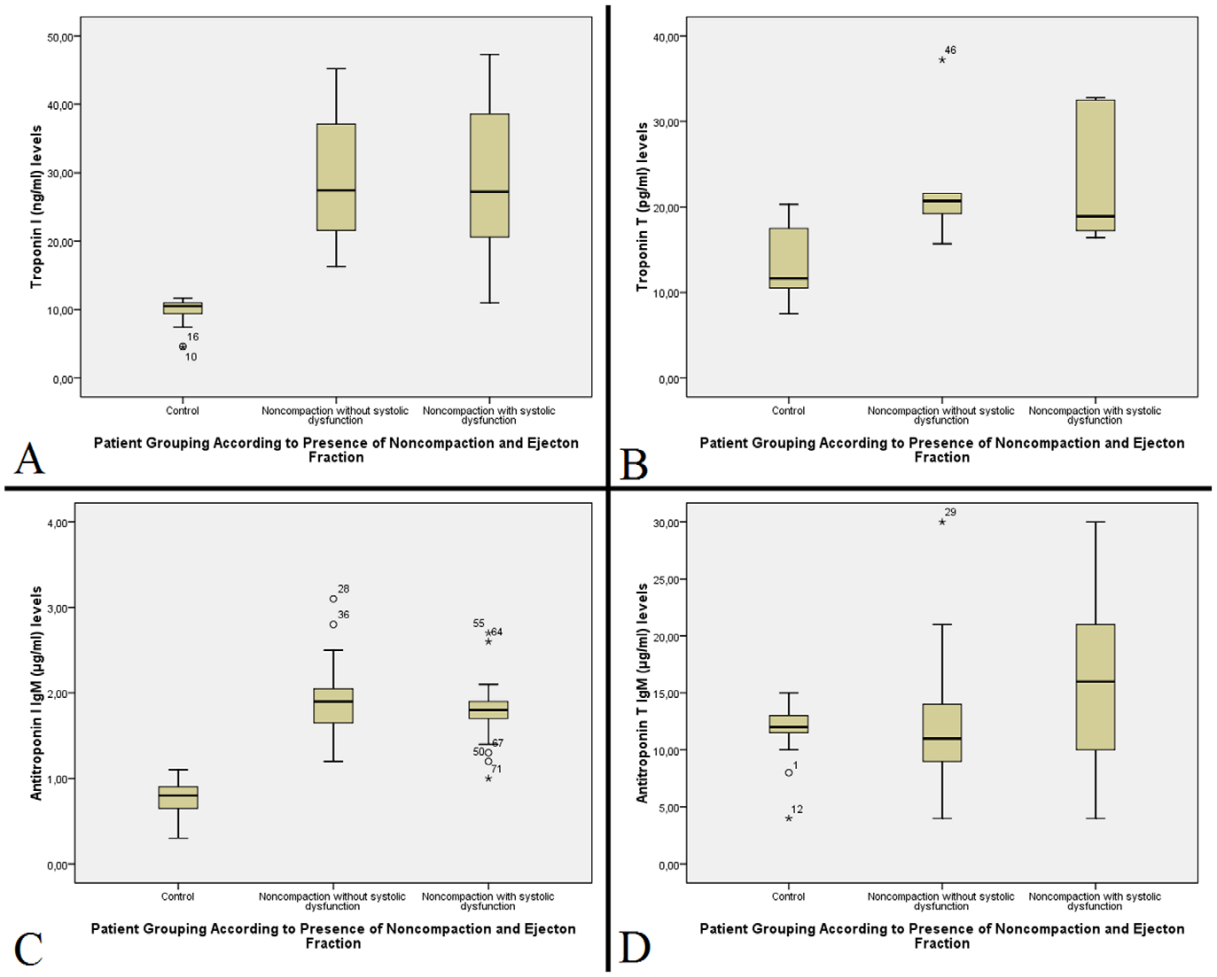
Boxplot diagrams showing troponin I, anti-troponin I IgM, troponin T and antitroponin T IgM levels in study groups. Troponin I (**A**), antitroponin I IgM (**B**) and troponin T (**C**) measurements were elevated in both NC/HT groups compared to controls, while antitroponin T IgM (**D**) levels were only elevated in subgroup of patients with reduced ejection fraction.

Both troponin T and troponin I levels were significantly higher in both NC/HT groups (22.17±6.97 pg/ml in NCNE group and 22.78±7.76 pg/ml in NCLE group for troponin T; 28.98±9.21 ng/ml for NCNE group and 28.11±10.42 ng/ml for NCLE group for troponin I) compared to controls (13.42±4.19 pg/ml for troponin T, p<0.01 and 9.74±1.97 ng/ml for troponin I, p<0.001). Anti-troponin I IgM antibodies were significantly higher in NC/HT patients (1.92±0.43 µg/ml for NCNE group and 1.79±0.36 µg/ml for NCLE group) compared to healthy volunteers (0.80±0.23 µg/ml for controls, p<0.001). In contrast, anti-troponin T IgM levels are only elevated in patients with noncompaction and systolic dysfunction (15.81±6.52 µg/ml) compared to patients in NCNE group (12.33±5.68 µg/ml, p<0.05) and healthy controls (12.00±2.32 µg/ml, p<0.05). Anti-troponin T IgG levels were similar to that of IgM levels (11.91±2.33 µg/ml for healthy controls, 12.34±5.80 µg/ml for NCNE group and 16.46±6.25 µg/ml for NCLE group, p = 0.01) (Figure 2).

**Figure 3:**
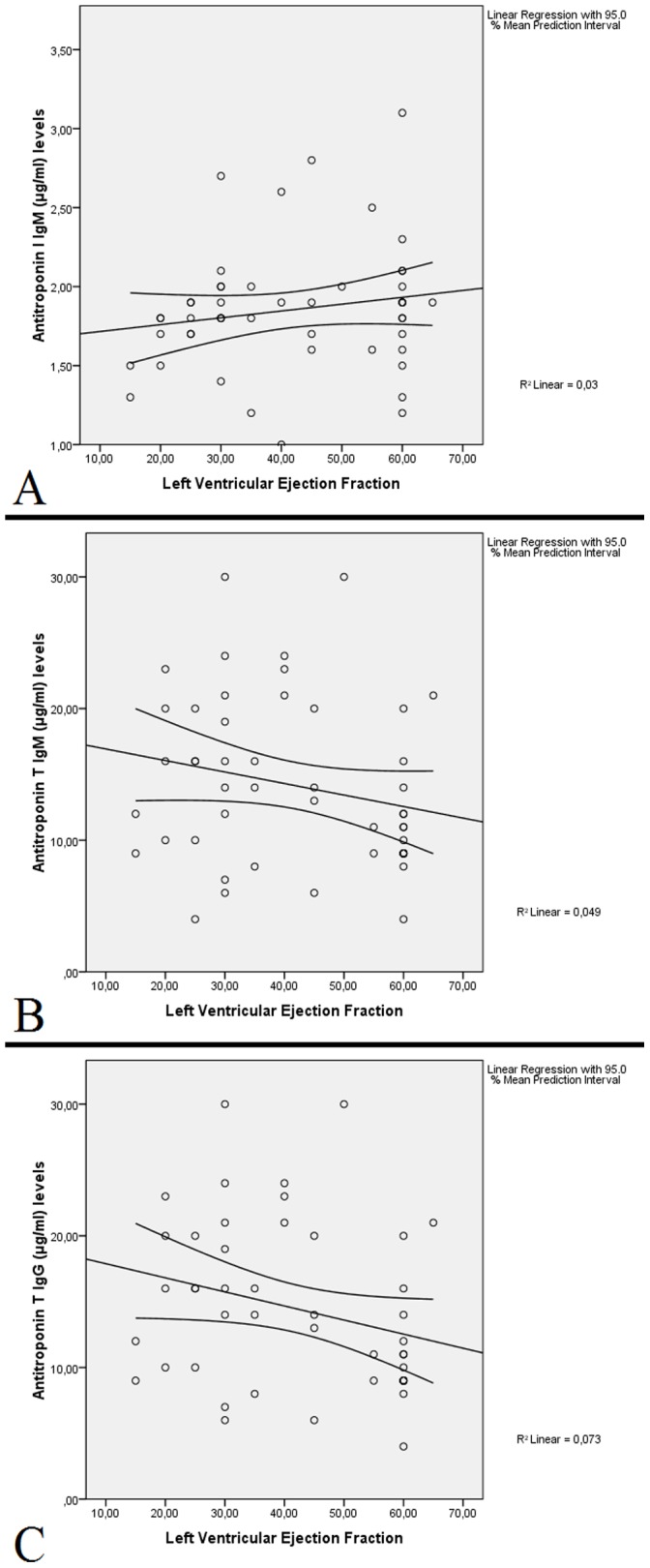
Scatter plot diagrams showing relationship between ejection fraction and antitroponin I IgM, antitroponin T IgM and antitroponin T IgG in noncompaction patients. Correlations for antitroponin I IgM (**A**), antitroponin T IgM (**B**) and antitroponin T IgG (**C**) did not reach statistical significance and had low correlation coefficients. Note that antitroponin T IgM levels were similar to antitroponin T IgG levels (in panels **B** and **C**).

Troponin I and antitroponin I IgM levels showed a significant correlation with each other (r = 0.696, p<0.001); while both parameters did not show a significant correlation with LVEF, LVEDv or LVESv in noncompaction patients. Troponin T did not correlate with antitroponin T IgM or IgG levels (r = −0.087, p>0.05 for IgM and r = −0.057, p>0.05 for IgG), and both parameters lacked a significant correlation with LVEF, LVEDv or LVESv in noncompaction patients (Figure 3). No significant correlation was observed between cTnI, cTnT, anti-cTnI IgM, anti-cTnT IgM or anti-cTnT IgG levels and number of segments with hypertrabeculation/noncompaction.

## Discussion

Cardiac troponins C, T and I are a part of troponin-tropomyosin complex, which control interaction of actin with myosin. Appearance of cardiac-specific troponins in circulation reflects myocardial damage. Chronic elevation of cardiac specific troponins is observed in conditions such as ischemic or dilated cardiomyopathies, presumably related with ongoing myocellular destruction [11]. Elevated troponin levels are almost invariably associated with poor prognosis in ischemic and dilated cardiomyopathy patients [12].

Our results show that both cardiac troponin I and cardiac troponin T were elevated in patients with NC/HT, regardless of initial systolic function. In patients with DCM, elevated troponin I and T are associated with myocyte injury or death and a progression of heart failure [4], [5], [12]. Therefore, increased troponin levels in study group suggest ongoing myocyte disruption or loss in NC/HT patients before and after reduction of ejection fraction. It is also noteworthy to recall that patients in NC/HT group and preserved ejection fraction had higher LVEDv and LVESv compared to controls, which could also indicate a progression from normal systolic function to dilated cardiomyopathy phenotype.

Anti-troponin antibodies are formed after exposure of troponins to circulation. They could be observed in very low quantities in normal individuals [6] and detected more frequently in patients after a myocardial infarction and dilated cardiomyopathy [8]. Experimentally, anti-troponin I autoantibodies are capable of altering calcium currents in cultivated myocardial cells and could produce “dilated cardiomyopathy-like” lesions [9]. In another study, exposure to anti-troponin I antibodies caused inflammatory myocarditis in mice [13]. Clinical studies had indicated a worse prognosis for patients with positive cTnI antibodies after an acute myocardial infarction [14]. Patients with iDCM who are positive for anti-cTnI antibodies had a trend towards increased left ventricular volume and heightened sympathetic activity [15]. In contrast, another study indicated that cell-mediated immune response against troponins could act to reduce immune response [16]. So far, only an association with anti-cTnI antibodies and cardiomyopathy could be demonstrated. Anti-cTnT antibodies were incapable of inducing myocardial damage, possibly due to sarcoplasmic location of cTnT [9].

We have shown that anti-cTnI IgM antibodies are elevated in patients with NC/HT, regardless of initial ventricular function. This finding is compatible with high cTnI levels in patients with NC/HT with or without reduction of ejection fraction. Since anti-cTnI antibodies are capable of inducing myocardial damage, it is possible that observed anti-cTnI antibody levels could initiate or progress systolic dysfunction in NC/HT patients. However, this hypothesis remains as a deduction and remains to be proven. In contrast, we observed that anti-cTnT IgM and IgG antibodies were elevated only in a subgroup of patients with NC/HT and reduced EF. Despite the fact that anti-cTnT antibodies could be produced after immunization with cTnT in mice, no damage to myocardial cells could be demonstrated [9]. As clinical trials were usually conducted with anti-cTnI rather than anti-cTnT, less information is available regarding to prognosis of anti-cTnT positive patients. While we observed higher anti-cTnT IgM and IgG in patients with NC/HT and reduced EF, no increase was observed in patients with NC/HT and normal systolic function. Based on previous experimental data, we assume that the effect of anti-cTnT antibodies on systolic dysfunction could be minimal.

Despite intense concern, left ventricular noncompaction remains an enigmatic disease. NC/HT can be observed in isolation or accompanying congenital anomalies or muscular dystrophies [17], [18]. This condition is thought to arise from an embryonic arrest in myocardial compaction process [19], while acquired NC/HT was also reported [20]. More importantly, the cause of ventricular dilatation and decline in systolic function is still not known. Our data shows that elevation in troponins and anti-troponin I autoantibodies, along with changes in ventricular geometry (increased end systolic and end diastolic diameters) precede reduction in systolic function. However, our data is insufficient to reveal the nature of ventricular damage before appearance of heart failure or whether autoimmunity plays a role in cardiac dysfunction.

### Study Limitations

This study is designed solely to investigate troponin and antitroponin antibody levels in patients with NC/HT. Our results should be interpreted with caution, as the presence of antibodies themselves does not imply a causal relationship between left ventricular dysfunction and immunity-mediated damage to myocytes. The causative role of antitroponin antibodies in the development of DCM is also debated.

A rise in antitroponin autoantibodies is not specific to NC/HT-related ventricular failure and was previously shown to be related with other causes of dilated cardiomyopathy [21]. Therefore, a rise in anti-troponin antibody levels could be anticipated in patients with reduced EF, regardless of initial etiologic factor. Nevertheless, our results did show a rise in autoantibody levels (for cTnI) before development of systolic dysfunction, which diverts our study with previously conducted studies on DCM.

Regrettably, we were unable to study anti-cTnI IgG antibodies, as we could not obtain necessary reagent. While anti-cTnT IgM and IgG levels were similar between groups, we could not verify whether anti-cTnI IgG levels followed a similar pattern with anti-cTnI IgM. While we expect a similar pattern between groups for anti-cTnI IgM and anti-cTnI IgG, as antigenic stimulation is continuous rather than intermittent, another study will be needed to confirm that.

### Conclusion

In conclusion, this is the first study that examines troponin levels and antitroponin autoantibodies in NC/HT. Our results show that both troponin I and T levels were elevated in patients with NC/HT, regardless of systolic function. Anti-troponin I levels were also elevated in both NC/HT groups, while antitroponin T levels were only elevated in patients with NC/HT and reduced EF. While these results point to ongoing myocardial damage and autoimmune response in NC/HT patients, further studies are required to ascertain the role of autoimmunity in NC/HT.
